# Adipose- and muscle-derived Wnts trigger pancreatic β-cell adaptation to systemic insulin resistance

**DOI:** 10.1038/srep31553

**Published:** 2016-08-16

**Authors:** Kamil Kozinski, Magdalena Jazurek, Pawel Dobrzyn, Justyna Janikiewicz, Katarzyna Kolczynska, Anna Gajda, Agnieszka Dobrzyn

**Affiliations:** 1 Laboratory of Cell Signaling and Metabolic Disorders, Department of Biochemistry, Nencki Institute of Experimental Biology, Polish Academy of Sciences, Warsaw, Poland; 2 Laboratory of Medical Molecular Biochemistry, Department of Biochemistry, Nencki Institute of Experimental Biology, Polish Academy of Sciences, Warsaw, Poland

## Abstract

Wnt signaling molecules are associated with obesity, hyperlipidemia, and type 2 diabetes (T2D). Here, we show that two Wnt proteins, WNT3a and WNT4, are specifically secreted by skeletal muscle and adipose tissue during the development of insulin resistance and play an important role in cross-talk between insulin-resistant tissues and pancreatic beta cells. The activation of Frizzled receptor and Wnt signaling in pancreatic islets via circulating WNT3a in blood resulted in higher insulin secretion and an increase in beta cell proliferation, thus leading to islet adaptation in a pre-diabetic state. Interestingly, in fully developed T2D, the expression profiles of *Wnt3a* and *Wnt4* in adipose tissue and muscle cells and blood plasma levels of these proteins were opposite to the pre-diabetic state, thus favoring the downregulation of Wnt signaling in beta cells and resulting in dysfunctional pancreatic islets. These results demonstrate that alterations in the secretion profile of a canonical Wnt activator (WNT3a) and inhibitor (WNT4) from insulin-resistant tissues during the development of T2D are responsible for triggering progression from a pre-diabetic to a diabetic state. We also show here that WNT3a and WNT4 are potent myokines, and their expression and secretion are regulated in response to nutritional and metabolic changes.

Type 2 diabetes (T2D) is one of the most common metabolic disorders, the prevalence of which is estimated to be about 171 million people worldwide, and this number is growing rapidly each year^1^. Obesity is the major predisposing factor for T2D. This disease is characterized by peripheral insulin resistance and pancreatic β-cell dysfunction^2^. During the pre-diabetic state, the body compensates for adipose and muscle insulin resistance through an adaptive increase in insulin secretion. This compensatory response of β-cells is achieved mainly through the expansion of β-cell mass and an increase in insulin secretion^3^. The ability of pancreatic β-cells to avoid hyperglycemia is a key factor in the prevention of T2D. β-cell mass in diabetic patients not only fails to expand but also significantly decreases^4^. Therefore, understanding the mechanisms that are responsible for sustaining pancreatic β-cell adaptation to peripheral insulin resistance is necessary for the long-term restoration of normoglycemia in T2D.

Genome-wide association studies have revealed several genomic loci that confer susceptibility to the development of T2D. At least 14 of these genes are implicated in pancreatic islet growth and function. Additionally, seven of them are either components or targets of the Wnt signaling pathway^5^. Genetic variations of the gene that encodes T cell-specific transcription factor 7-like 2 (TCF7L2) have been shown to be the most important T2D genetic risk factors in several human cohorts^6^. β-catenin/TCF7L2-dependent Wnt signaling (i.e., the canonical pathway) is involved in pancreas development, islet function, and insulin production and secretion^5^,^7^. The experimental loss of TCF7L2 function in islets and polymorphisms of *TCF7L2* alleles in humans impair glucose-stimulated insulin secretion (GSIS), suggesting that perturbations in the Wnt signaling pathway may substantially contribute to the susceptibility to T2D^8^. Furthermore, polymorphisms of the gene that encodes the Wnt pathway coreceptor (*LRP5*) have been associated with the obesity phenotype^9^. Missense mutations of *LRP6* have been linked to the risk of metabolic syndrome^10^.

Wnt proteins are secreted glycoproteins that bind specific members of the Frizzled (FZD) transmembrane receptor family on target cells. Wnts play essential roles as mediators of pancreas development and are capable of inducing pancreatic β-cell proliferation *in vitro* and *in vivo*^11^. Conditional knock-in of the active form of β-catenin in mice successfully promotes the expansion of functional pancreatic β-cells^12^, whereas knock-in of the potent Wnt inhibitor AXIN impaired the proliferation of neonatal β-cells^11^. Additionally, Wnt ligands stimulate insulin secretion *in vitro*^13^. The reduction of TCF7L2 levels by siRNA treatment in both isolated mouse and human islets decreased GSIS^14^,^15^,^16^. Furthermore, genetic ablation of *LRP5* caused an impairment in insulin secretion, thus underscoring the importance of Wnt signaling in pancreatic β-cell function^17^.

Recently, human adipocytes were shown to secrete Wnt signaling molecules that potently induced β-cell proliferation and insulin secretion *in vitro*^13^. Growing evidence suggests that circulating factors can regulate β-cell function in a paracrine-dependent manner. Considering both the proliferative properties of several Wnts and key role of adipose tissue as an endocrine organ^18^, one possibility is that Wnt ligands and Wnt signaling might contribute to cross-talk between insulin-resistant tissues and pancreatic islets. Up to date however, the effect of insulin resistance on Wnt proteins secretion in adipocytes and muscles was unknown. The present study investigated whether adipose- and muscle-derived Wnts contribute to pancreatic β-cell adaptation to systemic insulin resistance and the way in which the Wnt expression profile changes during the progression of type 2 diabetes.

## Results

### Insulin resistance affects expression and secretion of WNT3a and WNT4 in both adipocytes and myotubes

**Figure 1:**
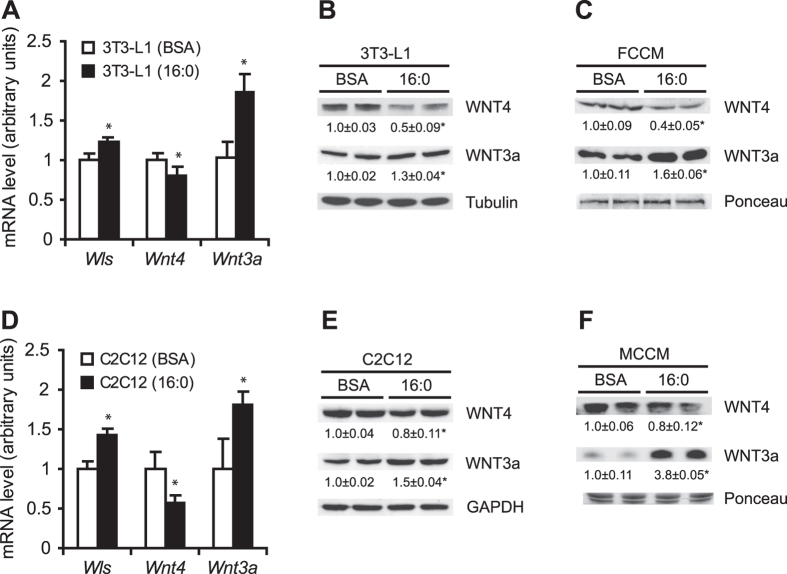
Effects of 16:0-induced insulin resistance on WNT3a and WNT4 protein levels and secretion in adipocytes and myotubes. mRNA and protein levels of WNT3a and WNT4 in control (BSA) and insulin-resistant (16:0) 3T3-L1 adipocytes (**A,B**) and C2C12 myotubes (**D,E**) were measured by real-time PCR and Western blot, respectively. The content of WNT3a and WNT4 was analyzed in FCCM (**c**) and MCCM (**f**) obtained from control BSA- and 16:0-treated cells. The data are expressed as mean ± SD, *n* = 3. *P < 0.05 vs. control (BSA).

Insulin resistance in 3T3-L1 adipocytes and C2C12 myotubes was induced by palmitate (16:0) treatment and confirmed by a decrease in AKT phosphorylation levels under insulin stimulation (Supplementary Fig. 1). To search for possible changes in the expression of genes that encode Wnt ligands, we analyzed a wide range of Wnt-related genes (i.e., *Sfrp1*, *Sfrp5*, *Wls*, *Wnt4*, *Wnt3a*, *Wnt5a*, *Wnt5b*, *Wnt6*, *Wnt7b*, *Wnt10a*, and *Wnt10b*), but only three of them (i.e., *Wls*, *Wnt4*, and *Wnt3a*) exhibited significant changes in mRNA levels in palmitate-treated 3T3-L1 adipocytes compared with control adipocytes (Fig. 1A). The expression of *Wls*, which encodes Wntless protein (i.e., a protein that is exclusively involved in the secretion of Wnt ligands^19^), increased by 20% in palmitate-treated adipocytes (Fig. 1A). The expression of the gene that encodes WNT4, a known inhibitor of the canonical Wnt signaling pathway^20^, decreased by 16%. The expression of the gene that encodes the canonical Wnt activator WNT3a increased by 80% in palmitate-treated 3T3-L1 adipocytes compared with bovine serum albumin (BSA)-treated cells (Fig. 1A). WNT4 and WNT3a protein levels decreased by 50% and increased by 30%, respectively, in insulin-resistant adipocytes compared with control cells (Fig. 1B). Importantly, changes in the gene expression and protein levels of *Wnt4* and *Wnt3a* were accompanied by changes in the rate of secretion of these proteins from insulin-resistant 3T3-L1 adipocytes. The level of WNT4 decreased by 60%, and the level of WNT3a increased by 70% in cell-conditioned medium from insulin-resistant 3T3-L1 adipocytes (fat cell conditioned medium [FCCM] 16:0) compared with the medium from control adipocytes (FCCM BSA; Fig. 1C).

Next, we detected *Wls*, *Wnt*, and *Wnt3a* expression and WNT4 and WNT3a protein levels in other insulin-sensitive cells (i.e., C2C12 myotubes). Interestingly, the profiles of *Wls*, *Wnt4*, and *Wnt3a* gene expression and protein levels in 16:0-treated C2C12 myotubes were similar to those observed for 3T3-L1 adipocytes compared with BSA-treated cells (Fig. 1D,E). The content of WNT4 decreased by 20%, and the level of WNT3a was almost 4-fold higher in cell conditioned medium from insulin-resistant C2C12 myotubes (muscle cell conditioned medium [MCCM] 16:0) compared with medium from control myotubes (MCCM BSA; Fig. 1F).

### FCCM from insulin-resistant adipocytes induces Wnt signaling, proliferation, and insulin secretion in β-cells via FZD receptor pathway

To investigate functional interactions between Wnts that are secreted by insulin-resistant adipocytes and β-cells in the context of pancreatic islet adaptation to systemic insulin resistance, we treated INS-1E cells and pancreatic islets that were isolated from rats with FCCM from palmitate-treated adipocytes and from control cells. Key elements of the canonical Wnt signaling pathway (i.e., the content of active [unphosphorylated] β-catenin and the activity of TCF7L2) were then analyzed. The WNT3a-conditioned medium from L-WNT3a cells was applied as a positive control.

**Figure 2:**
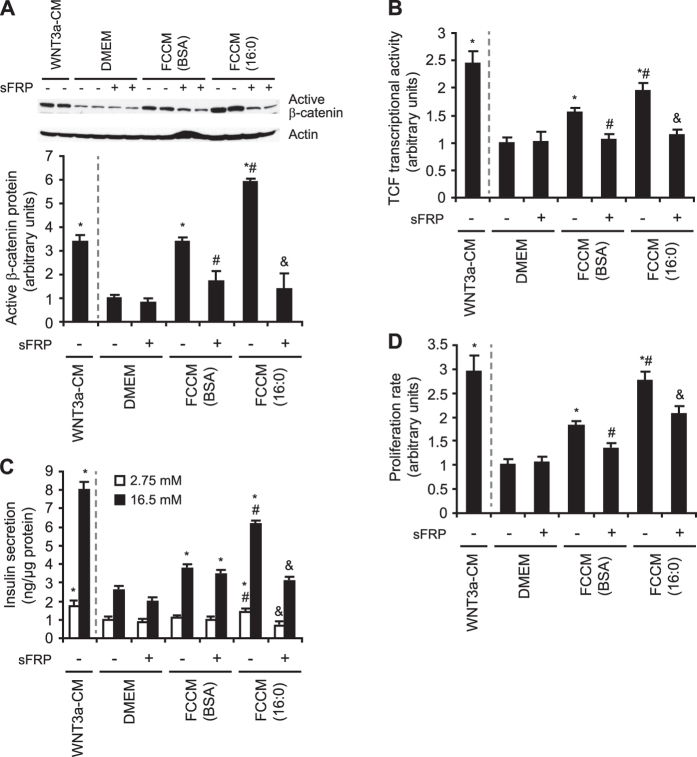
FCCM from insulin-resistant adipocytes induces Wnt signaling and the rates of insulin secretion and proliferation in INS-1E cells. (**A**) Active β-catenin protein level. (**B**) TCF7L2 transcriptional activity. (**C**) Insulin secretion under low (2.75 mM) and high (16.5 mM) glucose conditions. (**D**) Proliferation rate of INS-1E cells treated with FCCM BSA or FCMM 16:0. Luciferase activity (**B**) is expressed as relative light units. sFRP is a potent extracellular Wnt inhibitor. The WNT3a conditioned medium (WNT3a-CM) from L-WNT3A cells was applied as a positive control. The data are expressed as mean ± SD, *n* = 3. *P < 0.05 vs. DMEM, ^#^P < 0.05 vs. FCCM BSA, ^&^P < 0.05 vs. FCCM 16:0.

**Figure 3:**
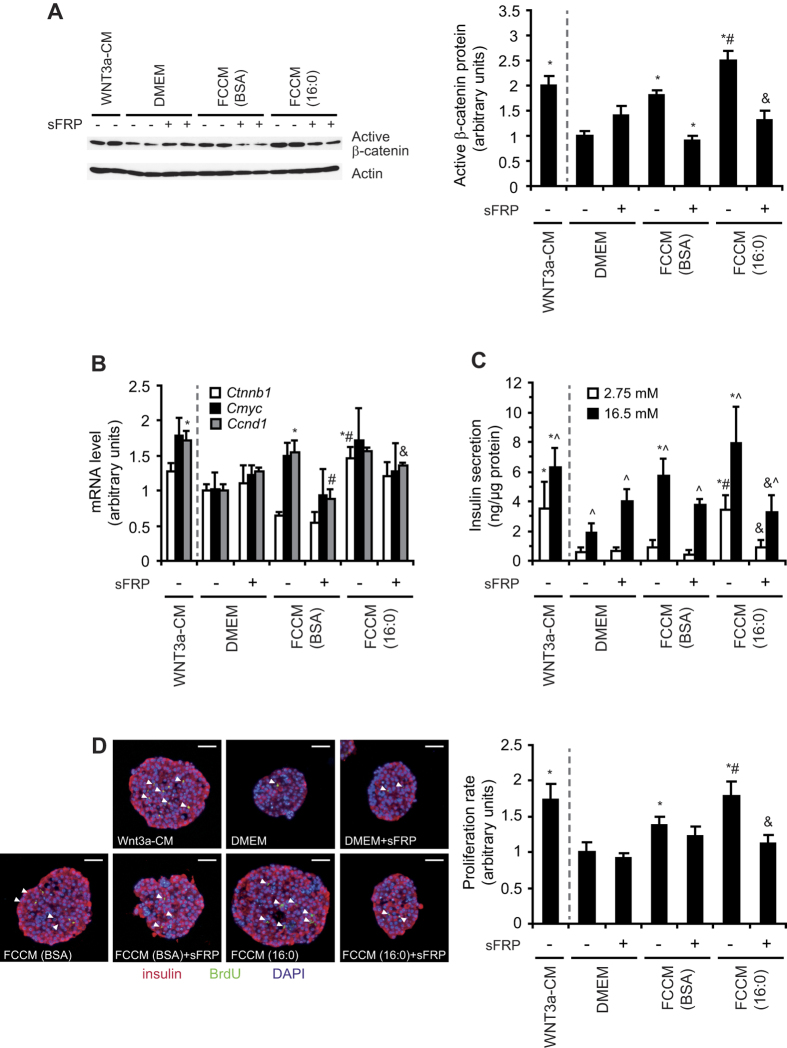
FCCM from insulin-resistant adipocytes induces pancreatic islet proliferation and insulin secretion. (**A**) Active β-catenin protein level in pancreatic islets was analyzed by Western blot. (**B**) The expression of Wnt signaling-related genes (*Ctnb1*, *Cmyc*, and *Ccnd1*) in islets was analyzed by real-time PCR. (**C**) Insulin secretion was measured under low (2.75 mM) and high (16.5 mM) glucose conditions and normalized to protein content. The data are expressed as mean ± SD, *n* = 3. *P < 0.05 vs. DMEM, ^#^P < 0.05 vs. FCCM BSA, ^&^P < 0.05 vs FCCM 16:0. (**D**) Proliferation rate of pancreatic islets, expressed as a quotient of BrdU dots and area of pancreatic islet section. The data are expressed as mean ± SD, *n* = 15. *P < 0.05 *vs*. DMEM, ^#^P < 0.05 vs FCCM BSA; ^&^P < 0.05 vs FCCM 16:0. sFRP is a potent extracellular Wnt inhibitor. The WNT3a conditioned medium (WNT3a-CM) from L-WNT3a cells was applied as a positive control.

FCCM from control adipocytes increased the level of active β-catenin 3.5-fold in INS-1E cells compared with cells that were treated with control medium (DMEM; Fig. 2A). However, in INS-1E cells that were treated with FCCM from insulin-resistant adipocytes, the level of active β-catenin was almost 6-fold higher compared with cells that were treated with DMEM and almost 2-times higher compared with cells that were treated with FCCM from BSA-treated adipocytes (Fig. 2A). An increase in the content of active β-catenin upon incubation with FCCM from insulin-resistant adipocytes was also found in rat pancreatic islets (Fig. 3A). To evaluate the influence of adipose-derived factors on Wnt signaling activity in β-cells, we analyzed the transcriptional activity of TCF7L2 using a luciferase reporter assay. The induction of the TCF7L2 reporter gene (TOP-flash) in INS-1E cells that were treated with FCCM from insulin-resistant adipocytes increased almost 2-fold compared with cells that were grown in fresh DMEM, whereas FCCM from BSA-treated adipocytes caused increased TCF7L2 activity by 50% (Fig. 2B). We also determined the expression of Wnt-related genes, including *Ctnnb1* (which encodes β-catenin) and two Wnt target genes (*Cmyc* and *Ccnd1*). Expression of the aforementioned genes was upregulated by FCCM treatment in both INS-1E cells (data not shown) and isolated rat islets (Fig. 3B), indicating higher TCF7L2 activity. Importantly, the effects of FCCM on the content of active β-catenin and transcriptional activity of TCF7L2 were abolished when secreted frizzled-related protein 1 (sFRP), a soluble Wnt antagonist, was applied to both INS-1E cells (Fig. 2A,B) and rat pancreatic islets (Fig. 3A,B), suggesting that the activation of Wnt signaling was mediated specifically by the FZD receptor pathway.

To evaluate whether the FCCM-induced activation of Wnt signaling affects the proper functioning of β-cells, we measured the rate of proliferation and insulin secretion in INS-1E cells and rat pancreatic islets. FCCM from BSA-treated adipocytes increased glucose (16.5 mM)-stimulated insulin secretion by approximately 40%. FCCM from insulin-resistant adipocytes increased glucose (16.5 mM)-stimulated insulin secretion more than 2.5-fold in INS-1E cells (Fig. 2C). Similar results were found in isolated rat pancreatic islets (Fig. 3C). The positive effect of FCCM (16:0) on the secretory capacity of INS1-E cells and pancreatic islets was inhibited by sFRP (Figs 2C and 3C). Similarly, FCCM (16:0) increased the proliferation rate of INS-1E cells (Fig. 2D) and β-cells in pancreatic islets (Fig. 3D). These effects were attenuated by sFRP (Figs 2D and 3D). These data suggest that FCCM from insulin-resistant adipocytes contains Wnt ligands more potently stimulate proliferation and induce the insulin secretion of β-cells via FZD receptors compared with FCCM from BSA-treated adipocytes.

### Wnt4 and Wnt3a expression in white adipose tissue (WAT) and skeletal muscle and alterations in the levels of these proteins in blood plasma during the progression of T2D

**Figure 4:**
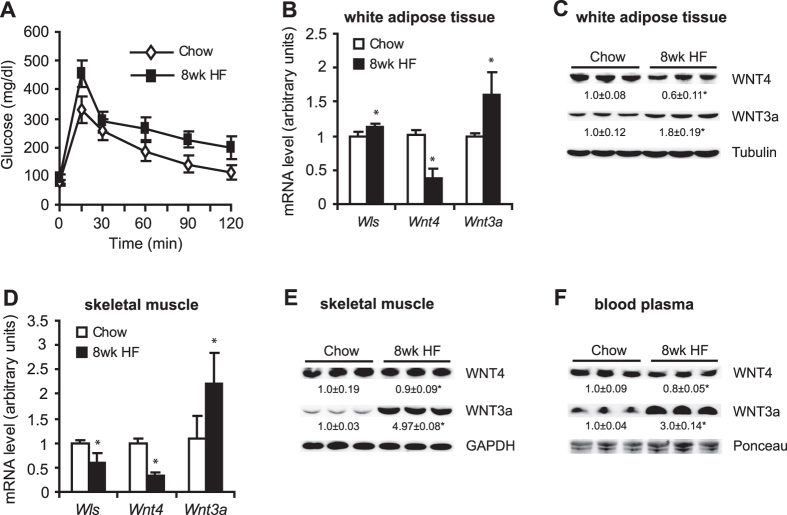
Gene expression and protein levels of WNT3a and WNT4 in WAT, skeletal muscle, and blood plasma in rats with pre-diabetes that was induced by 8 weeks of a HF diet. (**A**) Glucose concentration curves during glucose tolerance test. Gene expression of *Wls*, *Wnt3a*, and *Wnt4* in WAT (**B**) and gastrocnemius muscle (**D**) was measured using real-time PCR. The protein levels of WNT4 and WNT3a in WAT (**C**), gastrocnemius muscle (**E**), and blood plasma (**E**) in rats that were fed a HF diet for 8 weeks were analyzed by Western blot. The data are expressed as mean ± SD, *n* = 6. *P < 0.05 vs CHOW group.

**Figure 5:**
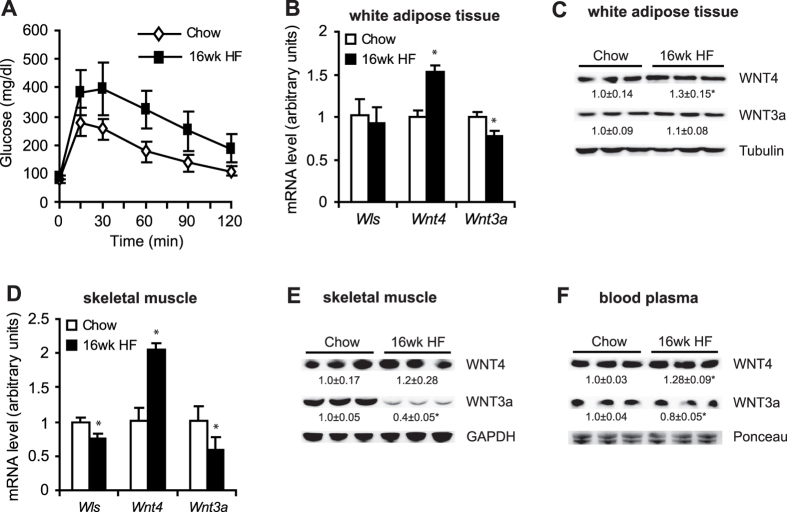
mRNA levels of *Wls*, *Wnt3a*, and *Wnt4* and protein levels of WNT3a and WNT4 in WAT, skeletal muscle, and blood plasma in rats with diabetes that was induced by 16 weeks of a HF diet. (**A**) Glucose concentration curves during glucose tolerance test. Gene expression of *Wls*, *Wnt3a*, and *Wnt4* in WAT (**B**) and gastrocnemius muscle (**C**). Protein levels of WNT4 and WNT3a in WAT (C), gastrocnemius muscle (**E**), and blood plasma (**F**) in rats that were fed a HF diet for 16 weeks. The data are expressed as mean ± SD, *n* = 6. *P < 0.05 vs CHOW group.

Wistar rats were fed a high-fat (HF) diet for 8 or 16 weeks to induce insulin resistance in peripheral tissues and T2D, respectively, determined by a glucose tolerance test (Figs 4A and 5A). In the pre-diabetic state (8-week HF diet), the expression of *Wnt4* was downregulated by approximately 60%, whereas the expression of *Wls* and *Wnt3a* was upregulated in WAT in the HF group compared with the group fed standard chow diet (CHOW) group (Fig. 4B). A significant reduction of *Wls* and *Wnt4* gene expression and a more than 2-fold increase in the level of WNT3a were found in gastrocnemius muscle after 8 weeks of the HF diet compared with the CHOW group (Fig. 4D). Changes in gene expression were paralleled by changes in WNT4 and WNT3a protein levels in both adipose tissue and skeletal muscle in pre-diabetic rats (Fig. 4C,E). WNT4 protein levels in plasma decreased by 20%, whereas WNT3a protein content was more than 3-fold higher in pre-diabetic rats compared with control animals (Fig. 4F).

Interestingly, in diabetic rats (16-week HF diet), the expression of *Wnt4* significantly increased, whereas *Wnt3a* significantly decreased in both WAT (Fig. 5B) and skeletal muscle (Fig. 5D) compared with the CHOW group. Additionally, WNT4 protein levels increased by 30% in WAT (Fig. 5C) and increased by 20% in skeletal muscle (Fig. 5E), whereas WNT3a protein levels were unchanged in WAT but decreased by 60% in skeletal muscle (Fig. 5C,E). Importantly, blood plasma protein levels of WNT4 increased by 28%, and WNT3a protein levels decreased by 20% in diabetic rats compared with the CHOW group (Fig. 5F).

### Activation of Wnt signaling is correlated with β-cell adaptation to systemic insulin resistance

**Figure 6:**
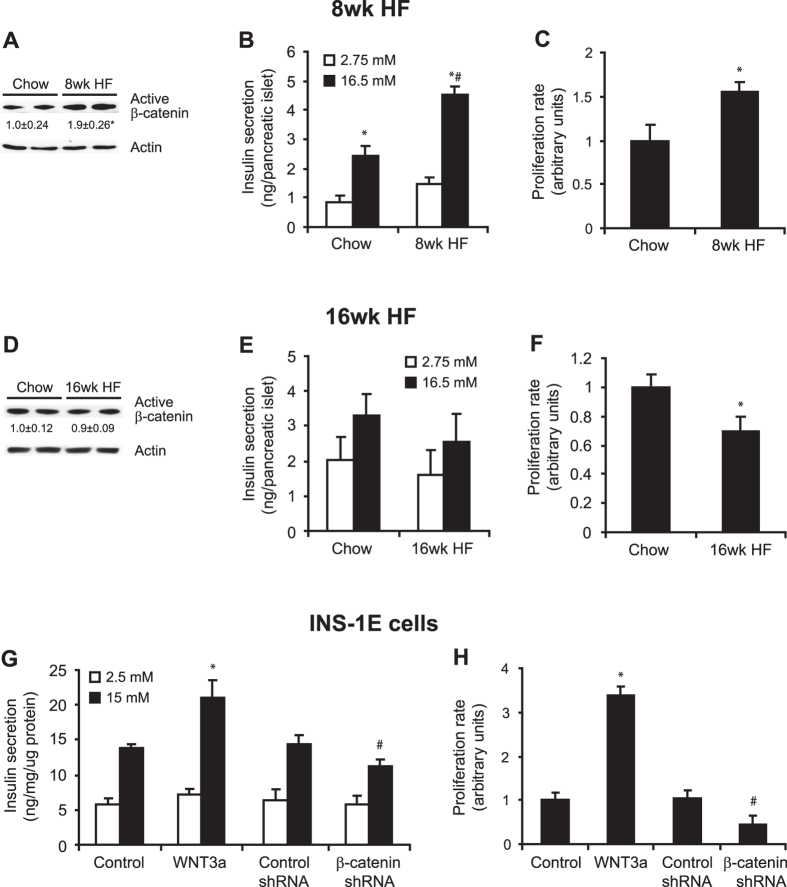
The activation of Wnt signaling is correlated with β-cell adaptation to insulin resistance. Active β-catenin content in pancreatic islets from rats that were fed a HF diet for 8 weeks (**A**) and 16 weeks (**D**) was measured by Western blot. Values were normalized to β-actin. The data are expressed as mean ± SD, n = 6. *P < 0.05 vs CHOW group. Insulin secretion in pancreatic islets from rats that were fed a HF diet for 8 weeks (**B**) and 16 weeks (**E**) was measured in under low glucose (2.75 mM) and high glucose (16.5 mM) conditions. The data are expressed as mean ± SD, n = 6. *P < 0.05 vs CHOW 2.75 mM glucose; ^#^P < 0.05 vs CHOW 16.5 mM glucose. The proliferation rate of β-cells in pancreatic islets from rats that were fed a HF diet for 8 weeks (**C**) and 16 weeks (**F**) is expressed as a quotient of BrdU dots and area of pancreatic islet section. The data are expressed as mean ± SD, n = 15. *P < 0.05 vs CHOW. The rate of insulin secretion (**G**) and proliferation (**H**) of INS-1E cells treated with shRNA against β-catenin or recombinant Wnt activator (WNT3a). The data are expressed as mean ± SD, n = 3. *P < 0.05 vs appropriate Control; ^#^P < 0.05 vs Control shRNA.

To investigate the way in which changes in the expression of Wnt ligands in insulin-resistant tissues and their levels in blood plasma affect the function of pancreatic β-cells in pre-diabetic and diabetic rats, we analyzed isolated islets. β-catenin-dependent Wnt signaling is involved in islet function via the regulation of β-cell proliferation and insulin synthesis and secretion^5^,^7^. 8 week of HF diet led to pancreatic islet adaptation as evidenced by increased insulin gene expression (Supplementary Fig. 2A), 8-times higher insulin content in the whole pancreas (Supplementary Fig. 2B) and increased fasting insulin level in blood plasma (Supplementary Fig. 2C). In contrast, in animals fed HF diet for 16 weeks, the fasting plasma insulin level, as well as insulin mRNA level and insulin content in pancreas were significantly reduced when compared with the 8wk HF group (Supplementary Fig. 2A–C). The level of active β-catenin was almost 2-fold higher in islets in pre-diabetic rats compared with the CHOW group (Fig. 6A). This was accompanied by a 3-fold increase in glucose-stimulated insulin secretion (Fig. 6B) and 60% increase in proliferation rate (Fig. 6C) in islets in pre-diabetic rats compared with the CHOW group. However, no differences were observed in the levels of active β-catenin between pancreatic islets that were isolated from diabetic rats compared with the CHOW group (Fig. 6D). Additionally, in islets from diabetic animals, insulin secretory capacity after glucose stimulation was no longer maintained (Fig. 6E) and their proliferation rate decreased by 30% compared with pancreatic islets that were isolated from their control counterparts (Fig. 6F). To evaluate a direct effect of Wnt inhibition/activation on islet functioning, we have performed experiments on INS1-E cells using shRNA against β-catenin and recombinant Wnt activator (WNT3a) to down-regulate or activate Wnt signaling, respectively. Down-regulation of Wnt pathway in β-cells caused drop in insulin-secretion (Fig. 6G) and significantly decreased the rate of cell proliferation (Fig. 6H), while Wnt activation resulted in opposite effects (Fig. 6G,H).

## Discussion

Wnt signaling molecules are associated with obesity, hyperlipidemia, and T2D^6^,^10^,^21^. The present study found that two Wnt proteins, WNT3a and WNT4, are specifically secreted by skeletal muscle and WAT during the development of insulin resistance and play an important role in crosstalk between insulin-resistant tissues and pancreatic β-cells. The activation of Wnt signaling in pancreatic islets via circulating WNT3a in blood resulted in higher insulin secretion and an increase in β−cell proliferation, thus leading to islet adaptation to the rising demands of insulin in a pre-diabetic state. These phenomena appeared to be mediated by FZD receptor signaling. We also found that WNT3a and WNT4 are potent myokines, and their expression and secretion can be regulated in response to nutritional and metabolic changes.

Adipose tissue and skeletal muscle are the main targets for the action of insulin and major contributors to insulin resistance in states of obesity. Both insulin and Wnt signaling play important roles in adipose development, the former by inducing adipocyte differentiation and the latter by inhibiting it^22^,^23^,^24^. Although these two pathways have opposite effects on adipose development, the insulin and Wnt signaling pathways are also known to exert convergent effects, such as their ability to inhibit glycogen synthase kinase 3β activity^25^. Furthermore, Wnt signaling has been shown to regulate the balance between myogenesis and adipogenesis and regulate insulin sensitivity in myoblasts^26^. In agreement with this, we found that in a state of insulin resistance that was induced by 16:0 and an 8-week HF diet, the canonical Wnt activator WNT3a was upregulated, whereas the expression of the Wnt inhibitor WNT4 significantly decreased in WAT and skeletal muscle, suggesting that WNT4 signaling counteracts WNT3a signaling in insulin-resistant tissues. These results are supported by a previous study that found that WNT4 suppresses canonical Wnt signaling that is mediated by the β-catenin/TCF7L2 complex and promotes myogenic differentiation in C2C12 cells, whereas WNT3A has opposite effects^27^. Thus, the higher *Wnt3a* expression and simultaneous downregulation of *Wnt4*, as reported herein, appear to be involved in the compensatory mechanism of lipid-induced insulin resistance in skeletal muscle and WAT. Interestingly, the present study showed that this mechanism disappeared after 16 weeks of a HF diet and the development of T2D.

Notably, in T2D and chronic insulin resistance, we observed *Wnt3a* downregulation and *Wnt4* upregulation in both skeletal muscle and WAT. Promoter analysis of transcription factors binding sites for *Wls*, *Wnt3a* and *Wnt4* genes revealed binding sites for transcription factors regulated by lipid species such as SREBP, RXR/LXR and RAR (*data not shown*). This could be a potential molecular link between HF diet and changes in expression of *Wnt* ligands. The other possible mechanism may involve epigenetic changes in promoters of *Wls*, *Wnt3a* and *Wnt4* genes in insulin-resistant cells and tissues as previously we have shown that palmitate and stearoyl-CoA desaturase, a key enzyme in palmitate metabolism, regulate gene expression by changing DNA methylation level^28^. This is consistent with previous studies that reported that the Wnt pathway was downregulated in WAT in mice that were fed a HF diet, db/db mice, and Zucker (fa/fa) rats, suggesting that chronic insulin resistance may lead to a reduction of TCF7L2 transcriptional activity^29^. These observations are also consistent with reports that TCF7L2 expression levels are significantly lower in omental and subcutaneous fat tissue in obese T2D patients compared with obese normoglycemic subjects^30^. In primary adipocytes and differentiated 3T3-L1 cells, short-term WNT3a treatment stimulated the expression of leptin mRNA via TCF7L2^29^, whereas long-term WNT3a treatment led to insulin resistance and a reduction of glucose uptake^31^. Whether WNT3a and WNT4 that are produced by adipocytes serve as “autocrine” factors to regulate TCF7L2 activity remains to be explored. However, the present study suggests that a reduction of Wnt activation in T2D may be one of the mechanisms that promote the progression of insulin resistance that is induced by a long-term HF diet, and that Wnt ligand expression can be nutritionally regulated in adipocytes and skeletal muscles.

**Figure 7:**
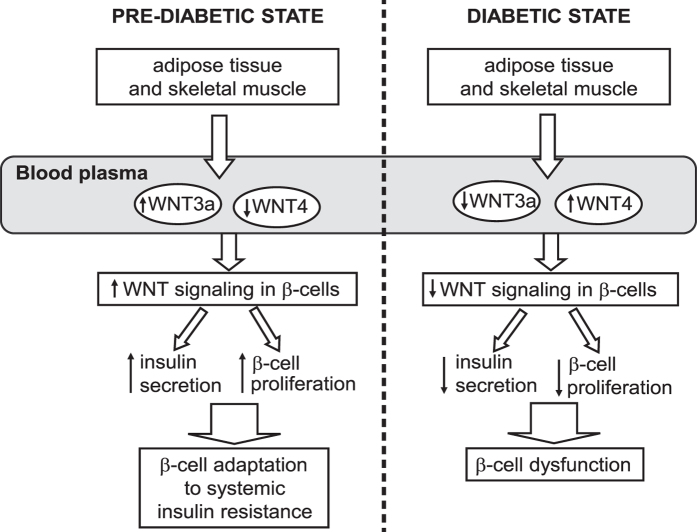
Wnt ligands contribute to β-cell adaptation to systemic insulin resistance. In the pre-diabetic state, WAT and skeletal muscle produce more activator (WNT3a) and less inhibitor (WNT4) of canonical Wnt signaling pathway, leading to the upregulation of Wnt signaling in pancreatic β-cells in an endocrine-dependent manner. Activated Wnt pathway triggers the adaptation of β-cells to systemic insulin resistance by increasing insulin secretion and β-cell proliferation. In the diabetic state (induced by 16-week HF diet), insulin-resistant tissues produce less WNT3a and more WNT4, which downregulates Wnt signaling in pancreatic β-cells. The reduction of Wnt pathway activation downregulates β-cell proliferation rate and insulin secretion, thereby negatively affecting β-cell adaptation to systemic insulin resistance and leading to pancreatic islet dysfunction.

All Wnt proteins share a signal sequence for secretion^32^,^33^. We found that higher gene expression and protein levels of WNT3a in insulin-resistant WAT and skeletal muscle resulted in higher WNT3a secretion from these tissues, and downregulation of the expression and protein levels of WNT4 was followed by a reduction of WNT4 secretion. Transfection of low amounts of the *Wls* plasmid was previously reported to be sufficient to promote WNT3a secretion^34^. Therefore, the higher expression of *Wls* that was observed in the present study may be responsible for the increase in WNT3a secretion by insulin-resistant tissues. We also found that Wnt ligands that were secreted from insulin-resistant tissues potently activated Wnt signaling in β−cells and promoted islet adaptation to insulin resistance via increasing β proliferation by adipose-derived factors was abolished by sFRP, demonstrating that this effect was specifically mediated by activation of the Wnt signaling pathway. These observations suggest a role for WNT3a and WNT4 that are secreted by insulin-resistant adipocytes and skeletal muscle as endocrine factors that contribute to islet adaptation to insulin resistance. Our data are consistent with Schinner and colleagues^13^, showing that human adipocyte-derived Wnt ligands can stimulate insulin secretion, glucokinase gene transcription, and β-cell proliferation *in vitro*. Our *in vivo* studies demonstrated that in the pre-diabetic state, the upregulation of *Wnt3a* expression and downregulation of *Wnt4* expression in insulin-resistant skeletal muscle and WAT, paralleled by changes in the levels of WNT4 and WNT3a in blood plasma, were responsible for activation of the Wnt signaling pathway in pancreatic β-cells in an endocrine-dependent manner. The accumulation of active β-catenin (i.e., the main element of the Wnt signaling pathway and indicator of Wnt activation) correlated with an increase in insulin secretion and β-cell proliferation in isolated pancreatic islets (Fig. 7). Prolongation of the HF diet for 16 weeks caused a severe diabetic state, in which β-cells no longer adapted to systemic insulin resistance. Interestingly, in this state, we found that the expression profiles of *Wnt4* and *Wnt3a* were opposite to those of the pre-diabetic state, favoring downregulation of canonical Wnt signaling. The lack of Wnt activation in pancreatic islets that were isolated from diabetic rats was correlated with downregulation of β-cell proliferation and deficiency in insulin production (Fig. 7). These results suggest that alterations in the secretion profile of a canonical Wnt activator (WNT3a) and inhibitor (WNT4) from insulin-resistant tissues during the development of T2D might be responsible for triggering progression from a pre-diabetic to a diabetic state.

In summary, our results reveal a novel mechanistic link between insulin resistance and hyperinsulinemia. The present data suggest that WNT3a and WNT4 are important components of crosstalk between insulin-resistant tissues and the pancreas in maintaining β-cell adaptation to systemic insulin resistance. In the pre-diabetic state, the activation of Wnt signaling correlated with β-cell adaptation to systemic insulin resistance. However, during the progression of T2D, the lack of Wnt activators (e.g., WNT3a) in systemic circulation may be responsible for β-cell failure. Therefore, sustained activation of Wnt signaling in pancreatic β-cells may be a promising target for the treatment of T2D.

## Materials and Methods

### Materials

The primary antibodies against AKT, the extent of phosphorylation of AKT at Ser473, and insulin were obtained from Cell Signaling Technology (Hertfordshire, UK). Peroxidase-conjugated β-actin antibody was purchased from Sigma (St. Louis, MO, USA). Antibodies against active form of β-catenin and against WNT3a were from Millipore (Billerica, MA, USA) whereas antibody against WNT4 were from R&D Systems (Minneapolis, MN, USA). FITC-conjugated anti-BrdU antibody was purchased from BD Biosciences (San Jose, CA, USA). Horse serum, fetal bovine serum (FBS), and DAPI reagent were obtained from Life Technologies (Carlsbad, CA, USA) whereas sFRP-1 was obtained from R&D Systems. The other chemicals were purchased from Sigma unless otherwise specified.

### Animals

Male Wistar rats, 6 weeks of age, were fed a HF diet that contained 60% calories from fat for 8 or 16 weeks (HF group, *n* = 6). The control group was fed a CHOW diet (CHOW group, *n* = 6). The animals were given *ad libitum* access to food and water. A glucose tolerance test was performed in CHOW- and HF-fed rats after overnight fasting, 1 week before sacrifice. At the end of the feeding period, the rats were anesthetized with pentobarbital and sacrificed. Blood was collected aseptically by direct cardiac puncture and centrifuged at 13,000 × *g* for 5 min at 4 °C to collect plasma. White WAT and gastrocnemius muscle were freshly isolated, frozen in liquid nitrogen, and stored at −80 °C. All experiments were performed in accordance with the approved guidelines and regulations and completed with the principles of laboratory animal care. All protocols were approved by the Ethical Committee for Animal Experiments at the Nencki Institute of Experimental Biology, Polish Academy of Sciences, Warsaw, Poland.

### 3T3-L1 cell culture and FCCM medium

3T3-L1 cells were cultured in Dulbecco’s Modified Eagle’s Medium (DMEM) with 10% bovine calf serum. Two days after reaching confluence, 3T3-L1 cells were treated with DMEM containing 1 mg/mL insulin, 0.25 mM dexamethasone, 0.5 mM 3-isobutyl-1-methylxanthine and 10% FBS for 3 days. Next, cells were maintained in DMEM supplemented with 1 mg/mL insulin and 10% FBS for another 3 days. Then medium was replaced with DMEM conditioned with 10% FBS for 1 day and after that insulin resistance was evoked by 24 h incubation with 1 mM palmitate (16:0) conjugated with bovine serum albumin (BSA)^35^. Control cells were incubated for the same period of time with a corresponding concentration of BSA. After 24 h of incubation, palmitic acid was removed from the 3T3-L1 culture and fresh DMEM was applied. Fat cell conditioned medium (FCCM) was collected after 72 h and stored in working aliquotes at −20 °C for further analysis. 3T3-L1 adipocytes were stimulated with 100 nM insulin for 10 min to assess their insulin sensitivity.

### C2C12 cell culture and muscle cell conditioned medium (MCCM)

Myoblasts were cultured in DMEM full medium (DMEM, 10% FBS, 2 mM L-glutamine, 100 μg/ml streptomycin, 100 IU/ml penicillin) at 37 °C in a 5% CO_2_ humidified atmosphere. Confluent myoblasts were allowed to fuse and differentiate into myotubes in DMEM-R medium (DMEM, 2% horse serum supplemented as described above). Myotubes were used 6 days after induction of differentiation. Insulin resistance was evoked by 24 h incubation with 0.5 mM palmitate. Control cells were incubated for the same period of time with a corresponding concentration of BSA. After 24 h of incubation, palmitic acid was removed from the C2C12 culture and fresh DMEM-R was applied. Muscle cell conditioned medium (MCCM) was collected after 72 h and stored at −20 °C for further analysis. C2C12 myotubes were stimulated with 100 nM insulin for 10 min to assess their insulin sensitivity.

### WNT3a conditioned medium from L cells (WNT3a-CM)

WNT3a containing medium (WNT3a-CM) was prepared from L cells that constitutively express WNT3a. DMEM medium with 10% FBS was conditioned for 3 days according to manufacturer’s procedures and stored in working aliquots at −20 °C.

### INS-1E cell culture and FCCM treatment

The rat insulin-producing INS-1E cells were grown in a monolayer at 37 °C with 5% CO_2_ in RPMI-1640 at 11 mM glucose supplemented with heat-inactivated 5% FBS, 10 mM HEPES, 50 μM 2-mercaptoethanol, 2 mM glutamine, 1 mM sodium pyruvate, 100 IU penicillin/ml and 100 μg streptomycin/ml until 80–90% confluence. All experiments were performed between passages 74 and 98. The cells were kindly provided by Prof. Pierre Maechler, Department of Cell Physiology and Metabolism, Medical Centre of the Geneva University.

FCCM was supplemented with 10 mM HEPES, 2 mM L-glutamine, 1 mM sodium pyruvate and 50 μM 2-mercaptoethanol just before experiment. Glucose concentration in the conditioned medium was adjusted to the equal values. sFRP-1 was added at a concentration of 0.6 mg/ml. INS1-E cells or pancreatic islets were incubated in FCCM for 24 hours. To activate Wnt pathway, the INS1-E cells were treated with 30 ng/ml of recombinant Wnt activator, WNT3a, (Sigma) for 24 hours.

### INS-1E insulin secretion assay

INS-1E cells were pre-incubated for 1 h at 37 °C in a glucose-free Krebs-Ringer bicarbonate buffer (135 mM NaCl, 3.6 mM KCl, 5 mM NaHCO_3_, 0.5 mM MgCl_2_, 10 mM HEPES, 1.5 mM CaCl_2_, 0.5 mM NaH_2_PO_4_, 0,1% BSA, pH 7.4). The buffer was then removed and the cells were incubated for 30 min at 37 °C in the same buffer containing 2.75 mM or 16.5 mM of glucose. At the end of the experiments, incubation media were removed and content of secreted insulin was analyzed using a Rat/Mouse Insulin ELISA Kit (Millipore). The amount of insulin release was normalized to the protein content.

### INS-1E proliferation test

After treatment of the cells with FCCM, media were removed and proliferation rate was analyzed using a cell proliferation ELISA, BrdU (Roche, Indianapolis, IN, USA), according to the manufacturer’s procedures.

### Islets isolation

Islets were isolated from Wistar rats by collagenase digestion and cultured as described previously^36^. Briefly, 7 ml of ice-cold 1.4 mg/mL collagenase solution was injected into pancreas *via* the common bile duct. The inflated pancreas was removed, transferred to a 50-ml conical tube containing 8 ml cold collagenase enzyme solution and incubated for 20–30 min at 37 °C. The islets were then purified by a Histopaque-1077 density centrifugation. Prior to initiating experiments, islets were rested overnight in RPMI-1640 medium supplemented with 10% heat inactivated FBS, 100 U/ml penicillin and 100 μg/ml streptomycin at 37 °C under a 95% air and 5% CO_2_.

### Islets insulin secretion assay

Islets were incubated in Krebs Ringer bicarbonate secretion buffer (25 mM HEPES, 114 mM NaCl, 23 mM NaHCO_3_, 5 mM KCl, 2 mM CaCl_2_ × 2H_2_O, 1, 2 mM MgSO_4_, 0, 1% BSA) containing 2.75 or 16.5 mM glucose at 37 °C for 45 min. At the end of incubations, aliquots of the incubation media were collected for insulin assay using a Rat/Mouse Insulin ELISA Kit (Millipore) according to manufacturer’s procedures.

### BrdU labeling

Pancreatic β-cells proliferation was evaluated by the measurement BrdU incorporation into cellular DNA. For these studies, islets isolated from experimental animals were incubated in 10 μM BrdU for 18 hours.

### Pancreatic islets immunohistochemistry

Pancreatic islets were fixed in Bouin’s solution for 2 h and maintained in 4% paraformaldehyde. The fixed tissue was embedded in paraffin and then cut into 5 μm thick sections. Indirect immunofluorescence was performed using rabbit anti-insulin antibodies. Direct immunofluorescence was performed using mouse FITC-conjugated anti-BrdU antibody. Nuclei were stained with DAPI reagent. Islets were photographed on the Olympus BX41 microscope.

### Western blot analysis

Protein extracts from INS-1E, 3T3-L1, C2C12 and pancreatic islets were prepared by lysing the cells with ice-cold lysis buffer containing 50 mM Tris-HCl pH 7.4, 1% igepal, 1% Triton X-100, 150 mM NaCl, 5 mM EDTA, 10 mM NaF, 1 mM phenylmethylsulfonyl fluoride, 1 mM NaWO_4_, 5 μg/ml pepstatin A, 10 μg/ml leupeptin, 1.4 μg/ml aprotinine. Visceral white adipose tissue and red gastrocnemius muscle were homogenized in ice cold buffer containing 0.5% Triton-X-100, 20 mM Tris, 2 mM EGTA, 2 mM EDTA, 2 mM NaWO_4_, 1 mM PMSF, 5 μg/ml pepstatin A, 10 μg/ml leupeptin, 1.4 μg/ml aprotinine. The protein concentrations were determined using the Bio-Rad protein assay (Hercules, CA, USA) with BSA as the standard. Protein levels of active β-catenin, WNT4, WNT3a, AKT and the extent of phosphorylation of AKT at Ser473 were determined in 50 μg of clarified homogenate protein. The separated proteins (10% SDS-PAGE gels) were transferred to Immobilon PVDF membranes (Millipore), which were blotted using appropriate antibodies. The proteins were visualized using Immobilon Western Chemiluminescent HRP Substrate (Millipore) as described by the manufacturer.

### Plasmid constructs

TOP-flash - luciferase reporter plasmid containing multiple copies of an optimal TCF-binding site was purchased together with FOP-flash (mutated plasmid) from Addgene (Cambridge, MA, USA). pRL-CMV - renilla luciferase plasmid was obtained from Promega (Madison, WI, USA). For knockodown experiments pLKO.1.sh.beta-catenin.2279, a vector containing shRNA sequence against β-catenin (Addgene plasmid # 19762), and pLKO.1 puro, an empty control vector for RNAi approach (Addgene plasmid # 8453), were used.

### Transient transfection

INS-1E cells were transiently transfected with 1 μg of TOP-flash or 1 μg of FOP-flash, together with 25 ng of pRL-CMV using Lipofectamine 2000 (Life Technologies) as described by the manufacturer. Luciferase reporter assay was performed according to the protocol of the dual-luciferase reporter assay system (Promega). The firefly luciferase activity is expressed as relative light units measured as quotient of TOP-flash and FOP-flash values, normalized to renilla luciferase acitivity. In shRNA-based gene knockdown experiments, 1 μg of plasmid containing shRNA against β-catenin (or control empty vector) and 2 μl of Lipofectamine 2000 were dissolved in 100 μl of RPMI -1640 medium. After 20 minutes to the transfection medium 400 μl of INS-1E cell suspension was added in an amount of 3 × 10^5^ cells per well. After 6 hours medium was replaced with RPMI-1640 complete medium. 72 hours after transfection cells were used in further experimental procedures.

### Gene expression analysis

Total RNA was isolated from INS1-E, C2C12, 3T3-L1 cells, pancreatic islets, visceral white adipose tissue and red gastrocnemius muscle using TRI reagent (Sigma). DNase-treated RNA was reverse transcribed with RevertAid H Minus First Stand cDNA Synthesis Kit (Life Technologies) and the real-time quantitative PCR was performed on a 7900HF Fast Real-Time PCR System (Applied Biosystems, Foster City, CA, USA). Fast SYBR Green Master Mix (Life Technologies) was used for detection and quantification of given genes, expressed as mRNA level normalized to a gene encoding β-actin or GAPDH by using the ΔΔC_t_ method. Primer sequences are available upon request.

### Statistical analysis

The results were analyzed using one-way analysis of variance followed by Bonferroni’s *post hoc* test. Values of *p* < 0.05 were considered statistically significant.

## Additional Information

**How to cite this article**: Kozinski, K. *et al*. Adipose- and muscle-derived Wnts trigger pancreatic β-cell adaptation to systemic insulin resistance. *Sci. Rep.* **6**, 31553; doi: 10.1038/srep31553 (2016).

## Supplementary Material

Supplementary Information
