# Supplementary material for: Adipose- and muscle-derived Wnts trigger pancreatic β-cell adaptation to systemic insulin resistance
Source: Sci Rep. 2016 Aug 16;6:31553. doi: 10.1038/srep31553 (PMC4985739; doi:10.1038/srep31553)
Supplement: Supplementary Information [file srep31553-s1.pdf]

Supplementary Materials

Adipose- and muscle-derived Wnts trigger pancreatic β-cell adaptation to systemic insulin resistance

Kamil Kozinski[1], Magdalena Jazurek[1], Pawel Dobrzyn[2], Justyna Janikiewicz[1], Katarzyna Kolczynska[1], Anna Gajda[1] & Agnieszka Dobrzyn[1]

[1]Laboratory of Cell Signaling and Metabolic Disorders, Nencki Institute of Experimental Biology, Polish Academy of Sciences, Warsaw, Poland

[2]Laboratory of Medical Molecular Biochemistry, Nencki Institute of Experimental Biology, Polish Academy of Sciences, Warsaw, Poland

Correspondence:

Agnieszka Dobrzyn

E-mail: adobrzyn@nencki.gov.pl

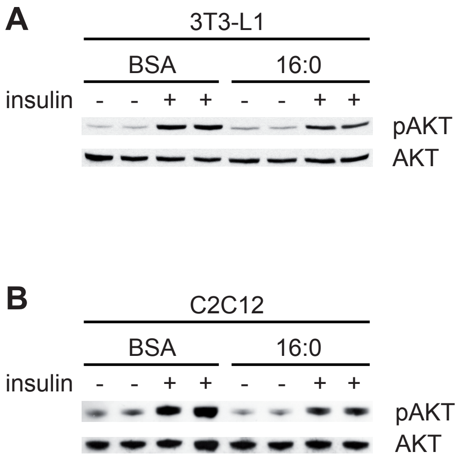

Supplementary Figure 1. Palmitate (16:0) treatment induced insulin resistance in 3T3-L1 (**a**) and C2C12 (**b**) cells. The protein level of AKT and extent of phosphorlation of AKT at Ser473 in BSA- and palmitate-treated cells were analyzed by Western blot.

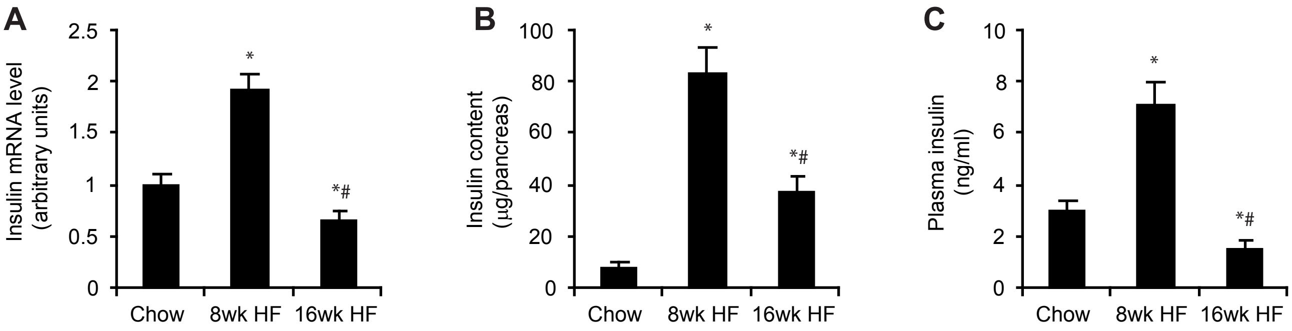

Supplementary Figure 2. Effect of high-fat feeding on insulin mRNA level in pancreas (**a**) and on insulin content in whole pancreas (**b**) and in blood plasma (**c**). Rats were fed high-fad diet for 8 or 16 weeks. mRNA level was measured by real-time PCR. Insulin content was measured using a Rat/Mouse Insulin ELISA Kit (Millipore), according to manufacturer's protocol. The data are expressed as mean ± SD, n = 6. *P < 0.05 vs CHOW group; #P < 0.05 vs 8wk HF group.
